# A Linear Framework for Time-Scale Separation in Nonlinear Biochemical Systems

**DOI:** 10.1371/journal.pone.0036321

**Published:** 2012-05-14

**Authors:** Jeremy Gunawardena

**Affiliations:** Department of Systems Biology, Harvard Medical School, Boston, Massachusetts, United States of America; Keio University, Japan

## Abstract

Cellular physiology is implemented by formidably complex biochemical systems with highly nonlinear dynamics, presenting a challenge for both experiment and theory. Time-scale separation has been one of the few theoretical methods for distilling general principles from such complexity. It has provided essential insights in areas such as enzyme kinetics, allosteric enzymes, G-protein coupled receptors, ion channels, gene regulation and post-translational modification. In each case, internal molecular complexity has been eliminated, leading to rational algebraic expressions among the remaining components. This has yielded familiar formulas such as those of Michaelis-Menten in enzyme kinetics, Monod-Wyman-Changeux in allostery and Ackers-Johnson-Shea in gene regulation. Here we show that these calculations are all instances of a single graph-theoretic framework. Despite the biochemical nonlinearity to which it is applied, this framework is entirely linear, yet requires no approximation. We show that elimination of internal complexity is feasible when the relevant graph is strongly connected. The framework provides a new methodology with the potential to subdue combinatorial explosion at the molecular level.

## Introduction

The overwhelming molecular complexity of biological systems presents a formidable scientific challenge. The mere number of protein-coding genes barely captures this complexity, [1]. Transcription factor binding to DNA to regulate gene expression and protein post-translational modification, to mention just two well-studied mechanisms, enable combinatorial construction of vast numbers of molecular states, [2]. How such complexity evolves and how it gives rise to robust cellular physiology are among the central questions in biology.

**Figure 1:**
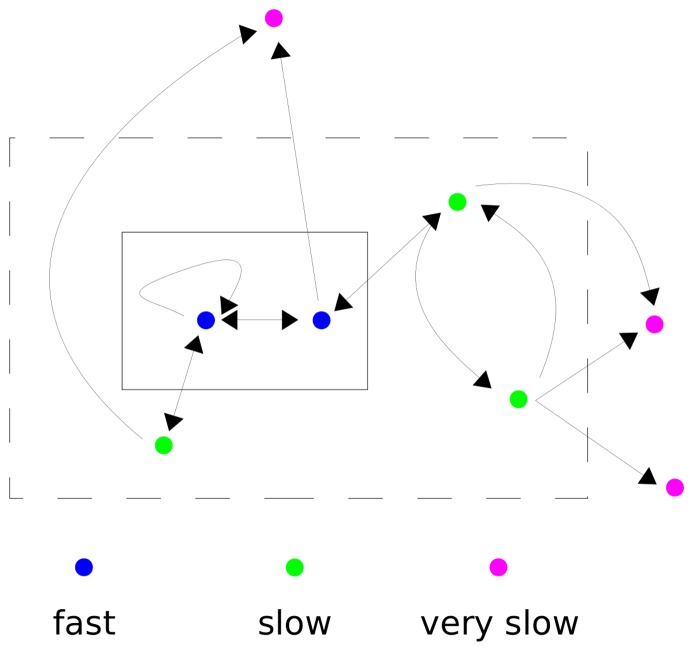
Schematic illustration of time-scale separation. A system is shown within the dashed box, which is assumed to contain all the components relevant to a given behaviour, so that it is partially uncoupled from its environment: it influences its environment (arrows leading outwards) but is not in turn influenced by the environment. Within the system is a smaller sub-system (box) which may be fully coupled to the larger system (bi-directional arrows). The components in the sub-system (blue dots) are taken to be operating sufficiently fast that they may be assumed to have reached a steady state, or a state of thermodynamic equilibrium, to which the remaining components in the larger system (green dots), and those in the environment that are influenced by the system (magenta dots), adjust on slower time scales. The Michaelis-Menten formula in (2) is derived from a time-scale separation of this kind.

One of the few conceptual methods for rising above this complexity, and thereby distilling general principles, has been time-scale separation (Figure 1). A system of interest (dashed box) is identified, which, for a particular behaviour being studied, is assumed to contain all the components relevant to that behaviour. A sub-system (box) within the larger system is taken to be operating sufficiently fast that it may be assumed to have reached a steady state or, as a special case of that, a state of thermodynamic equilibrium. The larger system and its environment adjust on slower time-scales to the steady-state of the sub-system. The components within the sub-system may be viewed as “fast variables”, while those additional components within the larger system are “slow variables”. Those components in the environment that might be influenced by the overall system are taken to be operating on the slowest time scale. Such assumptions often enable the internal states of the sub-system to be eliminated, thereby simplifying the description of the larger system’s behaviour.

Time-scale separation was first introduced at the molecular level in the famous work of Michaelis and Menten on enzyme kinetics, [3], [4]. They considered the following biochemical reaction scheme, in which an enzyme, *E*, reversibly binds to a substrate, *S*, to form an intermediate enzyme-substrate complex, *ES*, which then irreversibly breaks up to form the product of the reaction, *P*, and release the enzyme:
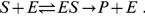
(1)A time-scale separation was assumed in which the free enzyme, *E*, and the enzyme-substrate complex, *ES*, were regarded as fast variables, while *S* and *P* were regarded as slow variables. (As a matter of historical accuracy, Michaelis and Menten made a simpler rapid equilibrium assumption. The so-called “quasi steady-state” assumption used here, and now universally employed, was first introduced by Briggs and Haldane, [5].) A simple algebraic calculation leads to the Michaelis-Menten rate formula
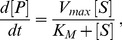
(2)in which the aggregated parameters 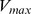 and 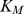 are determined by the underlying rate constants for the reactions in (1) and the total amount of enzyme that is present; see equation (9) below. Here, [X] denotes the concentration of the chemical species X.

This example has two characteristic features. First, the algebra has eliminated the fast variables, *E* and *ES*, leaving a formula that involves only the slow variable, *S*, on the right hand side. In this case, *P* does not appear on the right hand side because of the irreversibility of (1). Second, the expression on the right hand side is rational in the concentrations of the slow variables: it is a ratio of two polynomials in [*S*]. In this simple case, both numerator and denominator are first-order (linear) in [*S*].

Time-scale separations have been used in many distinct areas of biology, including enzyme kinetics, allosteric enzymes, G-protein coupled receptors, ligand-gated ion channels, gene regulation in both prokaryotes and eukaryotes and protein post-translational modification (see below). The characteristic features noted above, of elimination leading to a rational expression, are shared by all of them. Among these rational expressions are the familiar formulas of Michaelis-Menten in enzyme kinetics, Monod-Wyman-Changeux and Koshland-Némethy-Filmer in allostery and Shea-Ackers-Johnson in prokaryotic gene regulation. However, the corresponding analyses have been largely independent and *ad hoc*. In this paper we introduce a graph-theoretic framework that underlies all of these analyses. Despite the biochemical nonlinearity of the systems to which it is applied, the framework is entirely linear, yet it is not an approximation. Moreover, it is symbolic in the values of all rate constants, so that it may be used without having to know these values in advance. We show that elimination of internal complexity becomes feasible when the relevant graph is strongly connected and that, in this case, rational expressions emerge which can be explicitly described in terms of the graph. This procedure accounts for the rational expressions arising in all the time-scale separation examples just mentioned.

In these examples, time-scale assumptions have not always been justified by data. Instead, time-scale separation has been used as a hypothesis to simplify complex behaviour and thereby obtain new insights, which have subsequently guided conceptual understanding and experimental design. We follow the same practice here, treating time-scale separation as a method of mathematical simplification whose validity must ultimately be judged by its conceptual usefulness in guiding new experiments.

The examples mentioned above are reviewed next, to show the scope of application of the results and to point out some of the experimental insights. We then introduce the linear framework and explain how it underlies these different results. We also show how the framework appears in the study of general biochemical networks. Finally, as a demonstration of its power, we show how the framework can be readily adapted to deal with the more general situation in which a sub-system’s components are subject to synthesis and degradation. Applications of the framework to specific biological examples will be reported elsewhere.

## Results

### Examples of Time-scale Separation

#### Enzyme kinetics

Time-scale separation has been used to analyse enzymes with multiple substrates, products and effectors. As with the Michaelis-Menten formula in (2), the internal complexity arising from the bound states of the enzyme can be eliminated, leading to rational expressions involving only substrates, products or effectors. The algebra was formalised in the King-Altman procedure, [6], and the resulting rational expressions have been widely used in biochemistry, [7], [8]. For a modern example, see [9].

#### Enzyme allostery

Balancing supply and demand in metabolic networks requires enzymes to be controlled by molecules that are structurally distinct from the enzyme’s substrate. Allosteric regulation separates enzyme activity from control by exploiting distinct enzyme conformations having distinct activities. A molecule that binds preferentially to a conformation with weaker activity is an inhibitor, while one that binds preferentially to a conformation with stronger activity is an activator. Many enzymes subject to such regulation are oligomers with an axis of symmetry. A “concerted” model of enzyme allostery, in which the conformational changes were assumed to be limited to symmetric quaternary movements of the oligomer, was put forward in [10]. An independent “sequential” model, in which tertiary changes in the individual monomers were also allowed, was put forward in [11]. Subsequent formulations have allowed for additional complexity, [12], [13]. In each case, the enzyme conformations and ligand binding are assumed to be at thermodynamic equilibrium and the internal complexity arising from states with multiple bound ligands is eliminated. Enzyme activity is usually taken to be proportional to the equilibrium fractional saturation–the proportion of sites that are bound by ligand–leading to rational expressions such as the Monod-Wyman-Changeux formula, [10].

#### G-protein coupled receptors

GPCRs form the largest signalling superfamily in the human genome and one of the most clinically-significant, [14], [15]. Pioneering quantitative studies of the 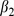-adrenergic receptor showed that apparent low- and high-affinity states of the receptor were explained by a “ternary complex model”, in which the receptor could be simultaneously bound to both a ligand and an accessory protein, later shown to be the G-protein, [16]. This basic model has been developed further to include distinct (allosteric) receptor conformations, [17], [18], and remains widely influential in quantitative pharmacology, [19], [20]. The possibility that distinct conformations recruit distinct subsets of the downstream signalling network, has been suggested as a basis for the phenomenon of collateral efficacy (“functional selectivity”, “protean agonism”, “stimulus trafficking”), [21], although post-translational modification of the receptor may also play an important role (see below). In these mathematical models of GPCRs, the conformational changes, ligand binding and accessory-protein binding are assumed to be at thermodynamic equilibrium and bound-states of the receptor are eliminated, leading to rational expressions for measures of downstream response.

#### Ligand-gated ion channels

Ion channels are oligomeric transmembrane proteins that regulate the movement of ions across the plasma membrane, [22], [23]. Ligand-gated ion channels have been investigated in exquisite quantitative detail by patch-clamp recording. The existence of distinct (allosteric) receptor conformations was suggested by an early model of the nicotinic acetylcholine receptor, [24]. This helped to distinguish the pharmacological properties of affinity and efficacy and similar models have since been widely used to understand quantitative channel behaviour, [25]. These models, like those for allosteric enzymes and GPCRs, assume that conformations and ligand binding are at thermodynamic equilibrium and thereby eliminate bound states of the receptor. Such equilibrium models have been adapted to yield discrete-state, continuous-time stochastic models of single receptors [26], from which new receptor conformations have been inferred, [27], [28]. These dynamic models show good agreement with experimental data, providing some justification for the assumption of thermodynamic equilibrium in this context.

#### Bacterial gene regulation

Gene transcription is regulated indirectly by the binding of transcription factors (TFs) to DNA. A model for expression of the lambda phage repressor was developed by Ackers, Johnson and Shea, in which TF binding was assumed to be at thermodynamic equilibrium and the net rate of gene transcription was treated as an average over the rates for the individual binding patterns. An implicit ergodic assumption is made that, under stationary conditions, the temporal frequency with which a pattern appears on a single molecule of DNA is the same as the normalised concentration of the pattern when TFs bind to many molecules of DNA. This “thermodynamic formalism” has been systematically developed for bacterial genes, [29], [30], and widely exploited in recent studies, [31], [32].

#### Eukaryotic gene regulation

Unlike prokaryotes, eukaryotic genes may be regulated by multiple transcription factors that can bind to multiple sites in widely-dispersed enhancer elements, giving rise to enormous combinatorial complexity, [1]. The thermodynamic formalism has also been used to analyse this more complex gene regulation, [33]–[35]. For instance, it has been used to determine how the Hedgehog morphogen in the *Drosophila* imaginal wing disc regulates the *patched* and *decapentaplegic* genes, [36]. In this and similar analyses, the rate of gene transcription is taken as the fractional occupancy of an additional binding site for an aggregated “basal transcriptional complex”. The thermodynamic formalism has also been tested in budding yeast using random promoter libraries driving fluorescent reporters, [37]. The formalism typically accounted for around 75% of the variance between different promoters, after taking into account inherent experimental variation, providing some justification in this context for the underlying time-scale separation.

#### Gene regulation away from thermodynamic equilibrium

Nucleosome repositioning can play an important role in eukaryotic gene regulation but is a dissipative process that cannot be treated at equilibrium. In a novel analysis, Kim and O’Shea went beyond the thermodynamic formalism to model regulation of the *PHO5* gene in budding yeast, for which the transcription factor Pho4 induces chromatin remodelling, [38]. A steady-state time-scale separation and an *ad hoc* calculation yielded a rational expression for the transcription rate as a function of Pho4 concentration that agreed well with experimental measurements.

#### Protein post-translational modification

Proteins with multiple types and sites of post-translational modification can exist in exponentially many global patterns of modification, or “mod-forms”. A protein with *n* sites of phosphorylation, for instance, has 2*^n^* potential phosphoryl-forms, providing another potent source of combinatorial complexity. Evidence from many sources reveals that distinct mod-forms may elicit distinct downstream responses. This was first seen in the PTMs that decorate the N-terminal tails of histone proteins, where distinct mod-forms guide differential assembly of transcriptional co-regulators, chromatin organisation and gene expression, giving rise to a “histone code”, [39], [40]. Such encoding has become a general theme relevant to many cellular processes, with the emergence of “co-regulator codes”, [2], “tubulin codes”, [41], and “GPCR barcodes”, [42], as reviewed in [43]. From a quantitative perspective, it is the “mod-form distribution”–the relative concentration of each of the mod-forms–that determines the functionality of a post-translationally modified protein. A mod-form with high influence on some downstream process, but at low relative concentration, may have less impact than one of low influence but high relative concentration. The overall influence of the protein is then an average over its mod-form distribution. Mass-spectrometric methods are now being developed to measure such distributions, [44]. The mod-form distribution is dynamically regulated by a continuous tug-of-war between the cognate forward and reverse modifying enzymes. This strongly dissipative process allows mod-form concentrations to be maintained far from equilibrium and to thereby transduce cellular information. Under very general conditions, the steady-state mod-form concentrations can be expressed as rational expressions in the enzyme concentrations–see equation (12) below–so that the effective complexity of the mod-form distribution depends, at steady state, only on the number of enzymes, not on the number of sites or on the types of modification or on the biochemical mechanisms of the enzymes, [45]. This enables average responses to be calculated, as in equation (13) below, and the behaviour of complex PTM systems to be analysed, without prescribing in advance either the number of sites or the rate constant values, [46]. The analysis developed in [45] was the starting point for the present paper.

#### Summary

The examples above fall into two broad classes, those considered at thermodynamic equilibrium, some of which have been treated by methods of statistical mechanics, such as the thermodynamic formalism in gene regulation, and those considered at steady state far from equilibrium, to which such methods do not apply. The framework introduced here integrates both classes and all the examples above. Thermodynamic equilibrium permits certain simplifications that are discussed below.

### The Linear Framework

**Figure 2:**
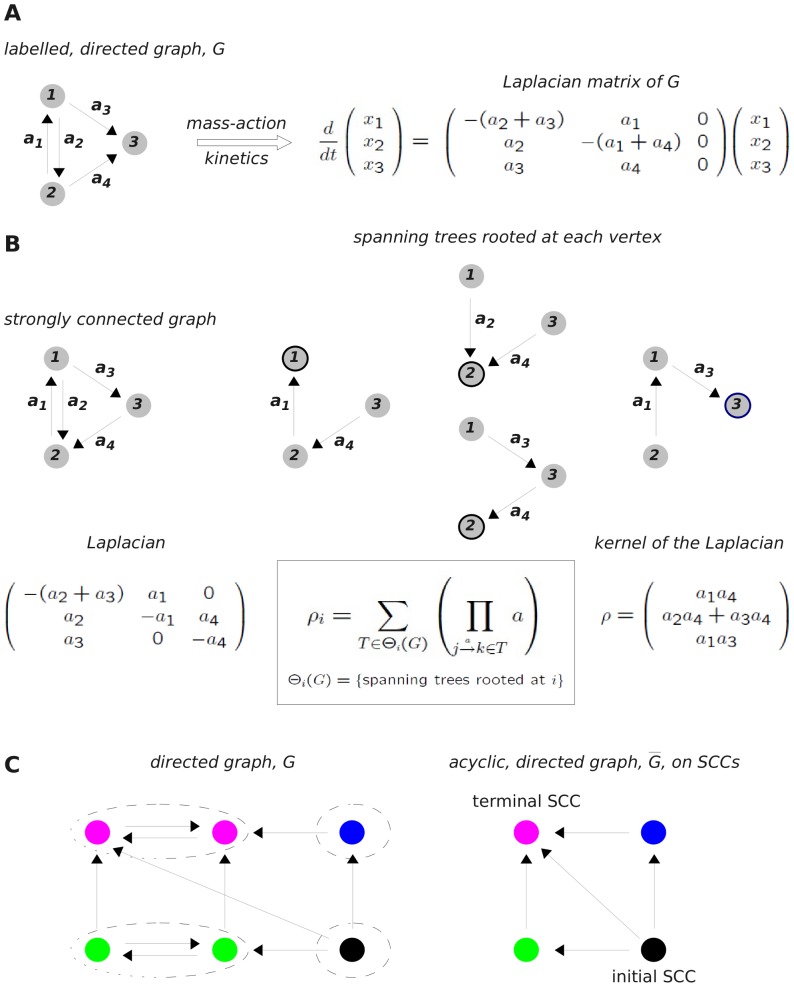
The linear framework. **A**. A labelled, directed graph, *G*, gives rise to a system of linear differential equations by treating each edge as a first-order chemical reaction under mass-action kinetics, with the label as rate constant. The corresponding matrix is the Laplacian of *G*. **B**. In a strongly connected graph (note the difference to the one in **A**), there are spanning trees rooted at each vertex, the roots being circled. The MTT gives an element of 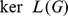 according to the formula in the box, as explained in the text. **C.** In a general directed graph, *G*, two distinct vertices are in the same strongly connected component (SCC) if each can be reached from the other by a path of directed edges. The SCCs form a directed graph, 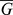, in which two SCCs are linked by a directed edge if some vertex of the first SCC has an edge to some vertex of the second SCC. 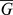 has no directed cycles, allowing initial and terminal SCCs to be identified.

We start from a graph, *G*, consisting of vertices, 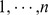, with labelled, directed edges, 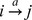, and no self loops, 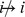 (Figure 2A). The vertices represent components of a system, on which a dynamics is defined by treating each edge as if it were a chemical reaction under mass-action kinetics, with the label as rate constant. We can imagine that an amount (or, equivalently, a concentration) of each component is placed on the corresponding vertex and that these amounts are transported across the edges in the direction of the arrows at rates that are proportional to the amounts on the source vertices. The constant of proportionality is the label on the edge. For instance, if the amounts of the components are denoted 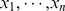, then the edge 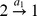 in Figure 2A contributes to the amount of component 1 at a rate 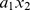, and so on. Since each edge has only one source vertex, the reactions are all first-order and the dynamics are therefore linear.

For the present, labels can be regarded as symbolic positive numbers with dimensions of 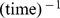. Positivity is not a restriction. A negative label sends the flux in the opposite direction and so has the same effect as reversing the direction of the edge and the sign of the label. It is in the interpretation of the labels that the leap can be made from the abstract linear system described here to a nonlinear biological system, as discussed in the next section.

The prescription above gives a system of linear, ordinary differential equations, which can be written in matrix form as
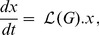
(3)where *x* is the column vector of component amounts and 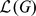 is called the *Laplacian matrix* of *G*. Such matrices were first introduced by Gustav Kirchhoff in his study of electrical circuits, [47]. They resemble discretisations of the continuous Laplacian operator but they are known in many different versions, [48]. As Laplacians have been widely studied, the results outlined here may be known under different guises.

Since material is neither created nor lost, the system has at least one conservation law given by the total amount of matter, 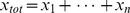, which remains constant at all times. Hence, 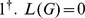, where 1 is the all-ones column vector and 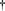 denotes transpose.

If we imagine the system being started with arbitrary amounts of each component, we expect that the dynamics will eventually relax to a steady state. This is, indeed, true for any graph. The dynamical behaviour of (3) has several interesting features, as well as biological applications; for instance, to the dynamical behaviour of ligand-gated ion channels. However, our interest here is in the steady state, so we defer a full discussion of the dynamics to elsewhere (I. Mirzaev, J. Gunawardena, in preparation). At steady state, 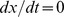, or, equivalently, *x* lies in the kernel of the Laplacian, 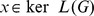. The kernel can be determined in two steps, first for a strongly connected graph and then for any graph.

A strongly connected graph is one in which any two distinct vertices can be joined by a series of edges in the same direction. The graph in Figure 2A is not strongly connected (vertex 1 cannot be reached from vertex 3), unlike that in Figure 2B. Strong connectivity depends only on the edge structure and not on the labels. A key observation is that, if the graph is strongly connected, then 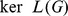 is one dimensional, [45]. No matter how many components are present in the graph and whatever arbitrary amounts of each component are present initially, once steady state is reached only a single degree of freedom is left. If the steady-state amount of any one component is known, then the steady-state amounts of all components are mathematically determined. This remarkable rigidity is the basis for the eliminations in all of the examples discussed here.

To actually calculate the steady states, it is necessary to determine a canonical basis element 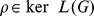. This is provided by the Matrix-Tree Theorem (MTT). Versions of this go back to Kirchhoff, [49], but the one needed for our purposes was first proved by Bill Tutte, one of the founders of modern graph theory, [50]. To calculate *ρ_i_*, take the product of all the labels on a spanning tree of *G* rooted at vertex *i* and add the products over all such trees (Figure 2B, box). A spanning tree is a fundamental concept in graph theory. It is a subgraph of *G* that contains each vertex of *G* (spanning) which has no cycles when edge directions are ignored (tree) and for which *i* is the only vertex with no outgoing edges in the tree (rooted). The spanning trees for the strongly-connected graph in Figure 2B are shown there along with the calculation of 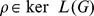. More spanning tree are shown in Supporting Information S1.

The kernel could have been calculated by standard linear algebraic methods using determinants. The significance of the MTT is that it expresses *ρ_i_* as a polynomial in the labels with positive coefficients (Figure 2B). The cancellations arising from the alternating signs in a determinant are thereby resolved.

Results equivalent to the MTT have been frequently rediscovered in biology, for instance, in the King-Altman procedure in enzyme kinetics, [6], and in Terrell Hill’s thermodynamical studies, [51], but without appreciating the broad scope of its application.

If *x* is any steady-state, then, since 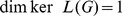, we know that 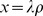, where 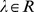. The undetermined 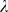 reflects the single degree of freedom that remains at steady state. It can be removed by normalising in different ways:
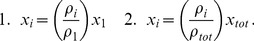
(4)In 1, one of the vertices, by convention vertex 1, is chosen as a reference. In 2, 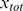 plays a similar role, with 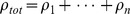.

It follows from the MTT that the terms in brackets in (4), 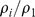 and 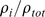, are rational expressions in the labels. The component amounts, *x_i_*, have been eliminated in favour of these rational expressions, along with *x*_1_ or 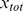, respectively. The rational expressions in each of the examples discussed here arise in exactly this way.

The situation when *G* is not strongly connected is also of interest. For a general graph *G*, the dimension of 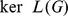 is given by the number of terminal strongly connected components. A strongly connected component (SCC) of a graph *G* is a maximal strongly-connected subgraph (Figure 2C). The SCCs themselves form a directed graph, 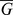, in which two SCCs are linked by a directed edge if some vertex of the first SCC has an edge to some vertex of the second SCC. 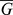 has no directed cycles, which allows initial and terminal SCCs to be identified. A description of 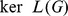 in terms of the terminal SCCs is given in the Appendix of [52]. We go further here by using the MTT to give explicit expressions for the basis elements in terms of the labels. For each terminal SCC, *t*, let 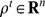 be the vector which, for vertices in that SCC, agrees with the values coming from the MTT applied to that SCC in isolation, while for any other vertex, 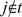, 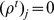. These vectors form a basis for the kernel of the Laplacian:
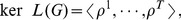
(5)where *T* is the number of terminal SCCs. An outline proof is provided in Supporting Information S1.

When *G* is not strongly connected there is more than one steady state, up to a scalar multiple. This should not be confused with multistability, as there is a corresponding increase in the number of conservation laws. Steady states may also have components with zero amounts, even when the initial conditions do not, in contrast to the strongly connected case, in which the MTT shows that each component is positive at steady state. For some applications, it is useful to know which steady state is reached from a given initial condition. Such dynamical issues will be dealt with elsewhere (I. Mirzaev, J. Gunawardena, in preparation).

We discuss some additional general results later but turn next to explaining how such a linear framework can be applied to nonlinear biological systems.

### The Uncoupling Condition

The leap from linearity to nonlinearity can be made in one of two ways. For time-scale separation, as in the examples discussed above, nonlinearity is encoded in the labels. Up till now, the labels have been treated as uninterpreted symbols. In any application the labels arise from the biochemical details of the system being studied. A label may be an arbitrary rational expression involving rate constants of actual chemical reactions or concentrations of actual chemical species. For instance, the following expression for a label would be legitimate
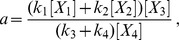
where 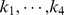 are rate constants and 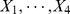 are chemical species. In our experience, so far, labels have been polynomials in the concentrations with coefficients that are rational in the rate constants. However, there is no mathematical reason to exclude more complex expressions, like the one above. The crucial restriction, which we refer to as the *uncoupling condition*, is that if a concentration, [*X*], appears in a label, then the species *X* must not correspond to a vertex in the graph. It could, however, correspond to a slow component in a time-scale separation, which is often how such a label arises. The uncoupling condition is essential to preserve linearity but it can be circumvented in some cases, as explained below. The other encoding of nonlinearity will be discussed later.

The key to applying the framework in a time-scale separation is, first, to find a directed graph whose components represent the fast variables in the sub-system, which has a labelling that satisfies the uncoupling condition, and, second, to show that the steady states of the linear Laplacian dynamics coincide with those of the full nonlinear biochemical dynamics of the sub-system. It is crucial to note that only the steady states need coincide, not the transient dynamics. If the latter were the same, then the sub-system would itself be linear, which is not the case in any of the applications. In this way, dynamical nonlinearity with simple rate constants is traded for dynamical linearity with complex labels. The trade-off is highly beneficial, as it allows the steady states of the nonlinear sub-system to be algorithmically calculated without knowing in advance the values of any rate constants. This enables the internal complexity of the fast components to be eliminated, giving rise to rational expressions based on one or the other of the normalisations in (4). This procedure underlies all the examples discussed here. We turn now to outlining how the framework is used in these applications.

### Applications Far from Equilibrium

#### Enzyme kinetics

As a simple demonstration of the framework, we return to the Michaelis-Menten example in the Introduction. More complex enzymes are treated in essentially the same way.

We first rewrite the reaction scheme in (1) after annotating the reactions with their corresponding rate constants:
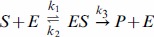
(6)The labelled, directed graph is constructed following the time-scale separation described in the Introduction. The vertices correspond to the fast components, which are the enzyme states *E* and *ES*. The edges amalgamate the effects of the reactions in which these components are involved. Edges outgoing from vertex *E* absorb the concentrations of slow components (in this case *S*), while all other edges only have rate constants in their labels. The following graph emerges
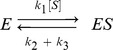
(7)in which the vertices have been annotated for convenience with the names of the corresponding components. [S] is the only concentration that appears in a label and it is not the concentration of a vertex in the graph. The uncoupling condition is therefore satisfied.

It is not difficult to check that, with this labelling, the steady states of the Laplacian dynamics given by (3) are the same as the steady states of the fast components given by the actual biochemical reactions in (6).

Graphs constructed like (7), even for more complex enzymes, are naturally strongly connected because bound states of the enzyme usually release the enzyme eventually. (If there are dead-end complexes, they must be formed reversibly, thereby also ensuring strong connectivity.) Accordingly, the MTT can be applied and it follows from the formula in Figure 2B that
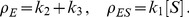
(8)If the system is assumed to be at a steady state, then it follows from the second elimination formula in (4) that



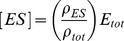
where 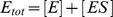 is the total concentration of enzyme and 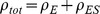. Note how [*ES*] has been eliminated in favour of 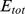 and the expressions appearing in *ρ*, which come from the MTT. The enzyme rate can now be calculated as



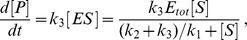
and the Michaelis-Menten formula in (2) emerges with the usual aggregated parameters, [8],



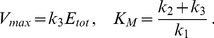
(9)King and Altman were the first to formalise this kind of algebra for more complex enzymes, [6]. They did not use graph theory and spanning trees but introduced “reaction patterns”, a terminology that has persisted in the biochemical literature, [8]. The King-Altman procedure is equivalent to the MTT.

#### Post-translational modification

The advantage of using the framework introduced here becomes particularly clear when moving from the behaviour of a single enzyme to that of a network of enzymes in post-translational modification. The identical mathematical machinery can be used in this quite different context. This was first described in [45] but we clarify here several issues whose significance was only understood more recently.

Consider a substrate, *S*, that is subject to different types of PTM (phosphorylation, methylation, acetylation, etc), with each modification potentially taking place at many sites. (There may be multiple such substrates but we consider only one for simplicity here.) We will analyse such a substrate under the assumption that it, and the enzymes acting upon it, are at steady state. The substrate *S* may have many mod-forms, which can be enumerated, 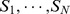. The number of mod-forms, *N*, typically depends exponentially on the number of sites, although, with modifications like methylation or ubiquitination, the detailed combinatorics may be more complicated, [43]. There is a natural directed graph on the vertices 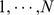, in which there is an edge 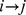 if some enzyme (and there may be several) is capable of converting *S_i_* to *S_j_*. Note that enzymes may be processive and able to make several modifications in one encounter between enzyme and substrate, so that an individual enzyme may be implicated in many edges with *S_i_* as the source vertex. It is an empirical observation that each individual transformation from *S_i_* to *S_j_* can generally be undone, if not directly, then through intermediate mod-forms. Hence, *S_i_* can be recovered, eventually, from *S_j_*, so that the directed graph is naturally strongly connected.

This is an obvious setting to apply the linear framework. A labelling of the edges is needed that captures the underlying biochemistry and also satisfies the uncoupling condition. PTMs fall naturally into two biochemical classes: those based on small-molecule modifications (phosphorylation, methylation, acetylation, etc) in which the donor molecules are regenerated by core metabolism and modification is undertaken by a single enzyme; and those based on polypeptide modifications (ubiquitin, SUMO, NEDD, etc) in which the donor molecules are synthesised by gene transcription and modification requires a series of enzymes, [43]. It is easiest to focus on the first class of small-molecule modifications; analysis of the second class is still work in progress.

With small-molecule modifications, the biochemistry of modification is significantly different from that of demodification, since the former involves the donor molecule that brings the modifying group, while the latter is a hydrolysis. Such details have been usually disregarded in the literature, where it has been the custom to treat all enzymes as if they followed the Michaelis-Menten scheme in (1). This overlooks much of the biochemical knowledge that has accumulated since 1913, [53]. Reaction mechanisms for kinases and phosphatases have been analysed, [54], [55], although less is known about the enzymology of other PTMs. One of the virtues of the linear framework is that it shows how realistic enzyme mechanisms can be analysed, thereby helping to bridge the gap between enzyme biochemistry and systems biology, [56]. By making appropriate assumptions regarding the core biochemical machinery that maintains the modifying groups, the modification and demodification enzymes may be assumed to follow mechanisms built up from just three basic reactions: creation of an intermediate complex; break-up of an intermediate complex; and conversion of one intermediate complex to another
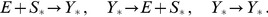
(10)Here, the intermediate complexes have been denoted 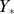, and the asterisk in 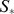 or 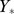 can be any index in the list of substrate forms or intermediate complexes, respectively. This scheme allows for complex enzyme mechanisms that may have multiple intermediates, yield multiple products, exhibit arbitrary degrees of processivity and be fully reversible. Enzymes may also use different mechanisms for different mod-forms. While (10) is quite general, it does impose some restrictions on enzyme mechanisms, as discussed further in [56], although these do not seem to be restrictive for most metabolic PTMs.

If the enzyme *E* is able to convert *S_i_* to *S_j_* by some mechanism built up from (10), then the linear framework may be applied to the enzyme intermediates just as described previously for enzyme kinetics. The intermediate complexes can be eliminated at steady state and the rate of conversion from *S_i_* to *S_j_* can be calculated as 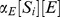, where 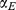 is a (possibly very complicated) rational expression in the rate constants of the mechanism. For instance, if the conversion from *S_i_* to *S_j_* takes place by the Michaelis-Menten scheme in (6), then it follows from (8) by using the first elimination formula in (4) that the conversion rate is given by
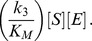
In this case, 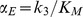. What the linear framework shows is that a similar formula holds for any reaction scheme built up from (10), no matter how complicated.

The key point is that such formulas are linear in [*S_i_*], which means that 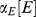 can be incorporated into a label in the mod-form graph. If several enzymes are able to convert *S_i_* to *S_j_*, each contributes additively to the rate, and the edge from *S_i_* to *S_j_* can be labelled
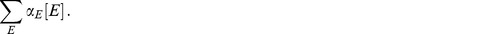
(11)The concentrations in such labels are those of enzymes. It follows that the uncoupling condition will be satisfied so long as enzymes are not also substrates. We assume this, for the moment, but explain below how it can be circumvented. With this labelling, it can be shown that the steady states of the Laplacian dynamics in (3) are identical with the steady states of the nonlinear dynamics coming from the specified enzyme mechanisms. This holds for arbitrary numbers of sites, arbitrary numbers of modifying and demodifying enzymes and arbitrary reaction mechanisms built up from those in (10), [45]. The MTT can now be applied to show that the substrate forms can be eliminated at steady state. The relative concentration of each mod-form, *S_i_*, can be explicitly written down using the second normalisation in (4),
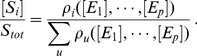
(12)We assume here that there are p forward and reverse enzymes in total, 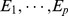. The ρi are given by the MTT as polynomials in the free-enzyme concentrations, through the labels described by (11). We see from (12) that the exponential complexity arising from combinatorial patterns of modification disappears at steady state: the effective (algebraic) complexity at steady state depends only on the number of enzymes, not on the number of sites. As we have seen, this surprising conclusion is a consequence of the strong connectivity of the mod-form graph.

In this example, the elimination process is hierarchical. The enzyme-bound intermediate complexes are first eliminated in favour of the mod-forms and the free enzymes, yielding the labels for the mod-form graph, and the mod-forms are then eliminated to leave only the free enzymes.

**Figure 3:**
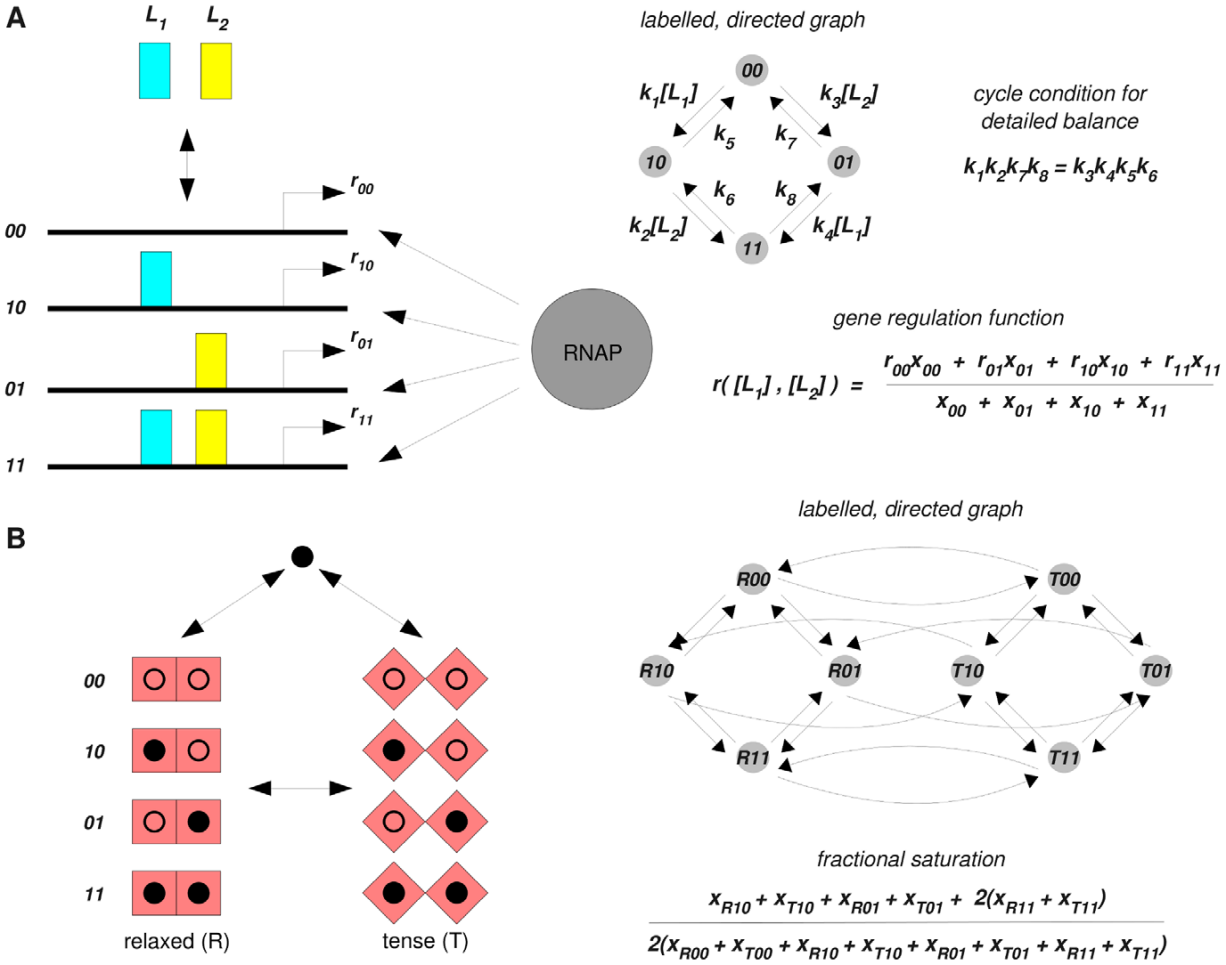
Ligand binding. **A**. Gene regulation, with two transcription factors (TFs), *L*_1_ and *L*_2_, binding to a promoter. A labelled, directed graph can be constructed as described in the text, with the microstates being denoted here by the bitstrings 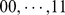. In this example there are no changes of conformational state in the scaffold, as would be the case if there was DNA looping or displacement of nucleosomes. The rate of mRNA transcription by RNA polymerase (RNAP) is assumed to depend on the pattern of TF binding as shown and the overall rate is calculated as an average over the probabilities of finding the promoter in each of the patterns. Assuming the system is at an equilibrium, *x*, these probabilities are given by the ratios, 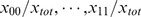, which can be calculated using the second elimination formula in (4). **B**. An allosteric homodimeric protein is shown in two conformational states, relaxed (R) and tense (T). The labelled, directed graph has both conformational state changes in the scaffold as well as ligand binding and unbinding. The microstates are denoted here 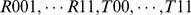. Labels have been omitted for clarity. For allosteric enzymes, the overall rate of product formation is usually assumed to be proportional to the fraction of sites that are bound by ligand (fractional saturation), as shown. This can be calculated as described in Supporting Information S1.

As discussed above, different mod-forms may elicit different downstream responses. The expression in (12) can be thought of as the steady-state probability of finding the substrate in mod-form *S_i_*. If *S_i_* elicits a quantitative level of effect given by 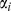, then the overall response of the PTM substrate can be estimated as an average over this probability distribution,
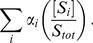
(13)It is assumed here, as part of the time-scale separation depicted in Figure 1, that the downstream response is operating sufficiently slowly to average over the different mod-forms. This is similar to the calculation of the rate of gene expression as a function of transcription factor concentrations (Figure 3A). In the case of PTM, formulas like (13) are functions of free enzyme concentrations, which are set by the enzyme mechanisms and cannot be readily estimated or approximated. However, since enzymes are assumed to be neither synthesised nor degraded (see below for how this restriction can be addressed), there is a conservation law for each enzyme. These may be explicitly written down in terms of the symbolic rate constants,
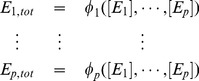
(14)to give *p* nonlinear algebraic equations for the *p* unknown free-enzyme concentrations. The essential nonlinearity in the biochemistry makes its appearance in these equations. For instance, for given total amounts of enzymes and substrate, there may be multiple solutions to (14), giving rise to multistability, [46], [57]. Equations (12) and (14) together provide a complete mathematical description of the substrate’s behaviour at steady state.

For PTM, the uncoupling condition has significant consequences, as it appears to rule out enzyme cascades, such as the MAP kinase cascade, in which the substrate at one level becomes the enzyme for the next. However, in this case, substrate-forms at distinct levels of the cascade can never be inter-converted and so appear in distinct connected components of the graph. If the concentration appearing in a label is that of a component in a different connected component, then, under suitable conditions, the analysis can still be undertaken in a recursive manner. Feliu *et al* work out the case of a linear cascade, [58], but the general conditions under which this method can be exploited have not yet been determined.

### Ligand Binding, at Equilibrium and Beyond

Several examples discussed above involve the binding of ligands to various kinds of “scaffolds”. These examples have usually been treated by assuming that the ligands and the scaffolds are at thermodynamic equilibrium. The case of the *PHO5* gene in yeast, discussed above, makes clear that a more general treatment is necessary. The linear framework is not limited to systems at equilibrium. We first describe how it can be used in sufficient generality to allow for dissipative behaviour and then discuss the simplifications that arise at equilibrium.

Consider a scaffold, *S*, that may correspond to an allosteric protein, receptor, ion channel, DNA segment, chromatin, etc. The scaffold can exist in multiple states, 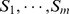, corresponding to protein conformations, states of binding of accessory proteins, states of DNA looping, patterns of nucleosome organisation, etc. The number of scaffold states need not be high; in the Monod-Wyman-Changeux model of allostery, for instance, *m* is often two, corresponding to a “relaxed” and a “tense” state (Figure 3B). The corresponding state transitions may take place at equilibrium, as in allosteric transitions, or require energy expenditure and be dissipative, as in nucleosome reorganisation. Ligands may bind to multiple sites on the scaffold in each of its states, with overlapping site preferences and cooperativity. To avoid excessive notation, we discuss here the case in which distinct ligands do not compete for sites, so that each site is associated with a specified ligand, which may or may not be bound there. In this case, the pattern of ligand binding can be encoded by a bitstring, 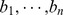, where *n* is the number of sites and 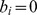 indicates that site *i* is empty, while 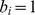 indicates that site *i* is bound by its cognate ligand. (A more complicated encoding is needed when distinct ligands bind to the same site.) The patterns of ligand binding in different scaffold states, or the “microstates” of the system, may then be encoded as
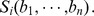
There are 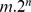 microstates in total although not all of these may be relevant, depending on the biological context.

The microstates form the vertices of the labelled, directed graph. The labelled edges arise from two sources. First, there may be transitions between scaffold states. These do not alter ligand binding and so take the form
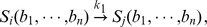
where *k*_1_ is the rate constant for the transition. Such transitions may or may not be reversible depending on the context. If the system can be treated at equilibrium, then all transitions must be reversible (see below). If not, some transitions may be irreversible. Second, there may be ligand binding and unbinding events. These do not alter scaffold state and so take the form



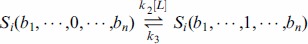
where *L* is the ligand. Here, only a single bit is changed. [*L*] is the steady-state, or equilibrium, concentration of the ligand *L* and *k*_2_ and *k*_3_ are the binding and unbinding rate constants, respectively. Examples of such labelled, directed graphs are shown in Figure 3A for gene regulation and in Figure 3B for allostery, using, for convenience, an abbreviated notation for the microstates.

This formulation is quite general. It allows for arbitrary degrees of cooperativity: the affinity of a ligand for a site may depend both on the conformation of the scaffold and on the occupancy of other sites.

Since the concentrations appearing in the labels are those of ligands, the uncoupling condition will be satisfied provided that none of the scaffold states can act as a ligand. This is the case in all the relevant examples discussed above. The situation in which the scaffold can aggregate may also be of interest, [12]. For the present, we assume that ligands and scaffolds are distinct, so that the uncoupling condition is satisfied. With this labelling, it is easy to check that the steady states of the Laplacian dynamics in (3) are the same as the steady states of the nonlinear dynamics arising from binding and unbinding of ligands and scaffold state transitions. Here, the nonlinearity arises only from ligand binding and trading it off into the labels is straightforward.

If the system is at thermodynamic equilibrium, then every edge must be reversible (see below). As long as the graph is connected (ie: does not fall into separate pieces with no edges between them), which is usually the case, it must necessarily be strongly connected. If the system is at steady state far from equilibrium, and some edges are not reversible, then strong connectivity must be confirmed. Strong connectivity allows the microstates to be eliminated in favour of the ligands, which, using the first normalisation in (4) and assuming there are *p* ligands, 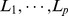, yields expressions like.
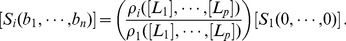
(15)Here, the reference microstate is taken to the be one in which no ligands are bound, which is helpful for calculating fractional saturation, as explained in Supporting Information S1. The second normalisation in (4) is more suitable for calculating rates of gene expression, following the method used previously for PTM.

As with PTM, the free-ligand concentrations are determined by *p* conservation laws for the *p* ligands, similar to those in (14). However, it is often assumed that ligands are in substantial excess over scaffolds, so that, to a first approximation, 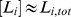. If this is not the case, then the *p* nonlinear equations must be solved to determine the actual free-ligand concentrations.

Strong connectivity is not mentioned in the dissipative analysis of *PHO5*, [38]. The corresponding gene regulation function was calculated by Matlab from the steady-state equations. In this example, the directed graph is essentially identical to that shown in Figure 4b of [38], which is easily checked to be strongly connected despite its irreversible edges. As we have seen, it is the strong connectivity that is essential for eliminating the microstates and calculating the gene-regulation function.

**Figure 4:**
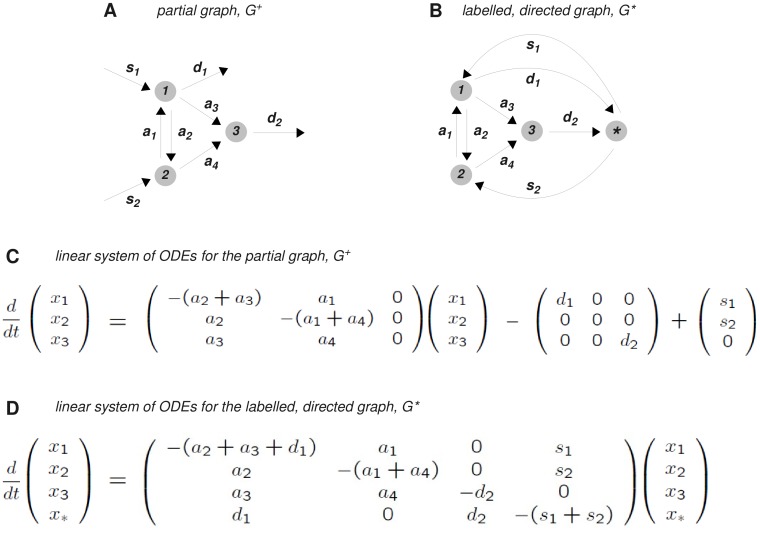
Synthesis and degradation. **A**. The non-strongly connected graph in Figure 2A is augmented with partial edges denoting synthesis and degradation to form the partial graph 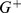 **B**. By introducing a new vertex, 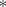, the partial graph 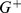 is transformed into the labelled, directed graph, 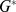, which, in this case, is strongly connected. **C**. The linear system of ODEs arising from the partial graph 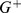. **D**. The Laplacian dynamics defined by the graph 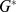. Since, in this case, 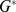 is strongly connected, the MTT can be applied to calculate the unique steady state of 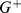, as given in equation (23). The details of the calculation are given in Supporting Information S1.

An important special case of the framework arises when the system is at thermodynamic equilibrium. In this case, the principle of detailed balance (DB) provides a simpler alternative to the MTT. The origins of this principle are discussed further in the next section. DB states that a chemical reaction at equilibrium is always reversible and that any pair of such reversible reactions is independently at equilibrium, irrespective of any other reactions in which the substrates or products participate. To see the implications for the labelled, directed graph constructed above, it is convenient to enumerate the microstates, as we did earlier when explaining the graphical framework, using the numbers 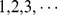 for the vertices and omitting the details of conformation and ligand binding. We assume the graph is strongly connected. DB implies that any edge 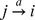 is reversible. Given any pair of reversible edges,
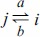
(16)and an equilibrium state *x*, DB implies that 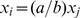, irrespective of any other edges impinging on *i* or *j*. The quantity 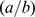 is an equilibrium constant.

DB makes it easy to calculate the equilibrium state, *x*. Choose a reference vertex, 1, which we assume to be a microstate in which no ligands are bound, such as 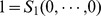. Given any other vertex *i*, choose a path of reversible edges from 1 to *i*,
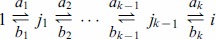
Such a path must always exist because of strong connectivity. It then follows that
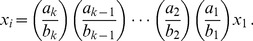
(17)This should be compared to (15) above. The product of equilibrium constants along the path corresponds to 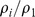. As before, this quantity is a rational expression in the labels but, now, the numerator and denominator consist only of the single monomials, 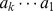 and 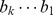, respectively. At equilibrium, DB cuts down the rooted trees of the MTT to a single path from 1.

Of course, there may be many such paths. However, the rate constants are not free to vary arbitrarily. DB requires that formula (17) gives the same result no matter which path is taken from 1 to *i*. This constraint may be summarised in the “cycle condition”: for any cycle of reversible edges, the product of the rate constants on clockwise edges equals the product on counterclockwise edges (Figure 3A). This condition is necessary and sufficient for (17) to be independent of the path taken and for every equilibrium state of the graph to satisfy DB; the proof is given in Supporting Information S1.

The so-called “thermodynamic formalism” has often been used to analyse systems at equilibrium, [59], [60], particularly in the context of gene regulation, [30], [35], [61]. The equilibrium state is calculated as a sum of interaction energies. The relationship between this and the linear framework comes through van’t Hoff’s formula for the equilibrium constant, which may be written, for the reversible edge (16),
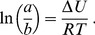
(18)Here, 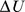 is the difference in Gibbs free energy between microstate *i* and microstate *j*, *R* is the gas constant (ie: the molar Boltzmann constant) and *T* is the absolute temperature. Formula (18) allows the multiplicative formulation of the linear framework in (17) to be converted to the additive formulation favoured in thermodynamics



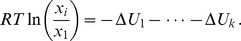
In gene regulation studies, the interaction energies have usually been limited to those of transcription factors binding to DNA and of transcription factors binding to each other when they are nearest neighbours or otherwise able to interact physically, [35], [37]. However, both the thermodynamic formalism and the linear framework can incorporate higher-order interactions and cooperativities as needed.

The thermodynamic formalism and the linear framework are equivalent for systems at thermodynamic equilibrium and related to each other as just explained. The linear framework comes into its own for analysing systems far from equilibrium and is well suited to the modern programme of unravelling complex eukaryotic gene regulation functions, [62].

### Chemical Reaction Network Theory (CRNT)

We mentioned previously that the linear framework can encode nonlinearity in two ways and we have discussed at length the first way, through the labels. Here, we briefly discuss the second way, through the vertices, which arises in CRNT. In this case, the framework is not associated with a time-scale separation but CRNT is often used to determine steady-state properties.

CRNT originates in Horn and Jackson’s pioneering attempt to extend thermodynamic reasoning from equilibrium to far-from-equilibrium systems, [63]. If we have a reversible chemical reaction, such as
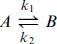
then mass-action kinetics implies that the ratio of the equilibrium concentrations is independent of the starting conditions of the reaction and depends only the rate constants,
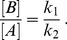
(19)A similar relationship holds generally for any chemical reaction at equilibrium. Such formulas can also be deduced directly from equilibrium thermodynamics, without making kinetic assumptions about the rates of reactions, [64]. For an isolated reaction such as this, kinetics is consistent with thermodynamics. However, a network of chemical reactions may have kinetic equilibria that do not satisfy thermodynamic constraints. Gilbert Lewis sought to avoid such paradoxes by suggesting the principle of detailed balance (DB), as stated in the previous section, [65]. It was later realised that DB is a consequence of the fundamental time-reversibility of microscopic processes, whether classical or quantum, [66], [67].

Horn and Jackson sought to extend thermodynamic properties like (19) to steady states far from equilibrium, [63]. Under mass-action kinetics, any network of chemical reactions gives rise to a system of nonlinear ordinary differential equations, 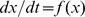, for the concentrations, 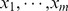 of the various chemical species. Here, the nonlinear function 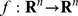 defines the dynamics on the species level. To disentangle the nonlinearities, the stoichiometric expressions that appear on either side of a reaction were treated as new entities called “complexes”. A hypothetical reaction such as 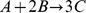 gives rise to the two complexes, 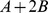 and 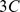. Each reaction defines a directed edge between complexes and the mass-action rate constant provides the edge with a label. Any network of chemical reactions, *N*, thereby gives rise to a labelled, directed graph, *G_N_*. The number of vertices in *G_N_* is the number of distinct complexes, *m*, among all the reactions in the network.

The Laplacian matrix of this graph defines a linear function, 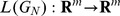, at the complex level that is the analogue of the nonlinear 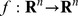 at the species level. The relationship between *f* and 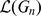 is expressed in the fundamental equation.
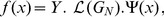
(20)where 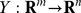 is a linear function that records the stoichiometry of each species in a complex and 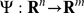 is a nonlinear function that records the mass-action monomial corresponding to each complex, [63]. The “dot” signifies composition of functions. Horn and Jackson were unaware at the time of the Laplacian interpretation, which was first pointed out in [68]. The decomposition in (20) is the starting point of CRNT, which was subsequently developed by Feinberg and his students, [69], [70]. The decomposition leads to Horn and Jackson’s concept of a “complex-balanced” steady state, *x*, for which 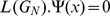. This may be reached far from equilibrium but still satisfies properties to be expected at equilibrium, including relationships between concentrations and rate constants that generalise (19), [63].

### Synthesis and Degradation

A common feature of all the examples discussed previously is that synthesis and degradation were entirely ignored, although they are often significant in the biological context. It is an indication of the power of the linear framework that it can be readily extended to accommodate this.

Consider, as before, a labelled, directed graph, *G*, on vertices 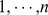 but now allow each vertex in this “core graph” to have additional partial labelled edges,
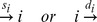
corresponding to zero-order synthesis or first-order degradation, respectively (Figure 4A). Each vertex may have any combination of synthesis and degradation, including neither or both. Call this “partial graph” 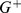. As before, there is a linear dynamics on 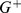, which may be described by the system of differential equations
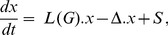
(21)where L(G) is the Laplacian matrix of the core graph, G. Here, 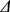 is a diagonal matrix with 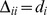 and S is a column vector with 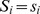, using the convention that di or si is zero if the corresponding partial edge at vertex i is absent.

The dynamics defined by (21) has several different features to that described by (3). It is easy to construct examples in which degradation cannot keep up with synthesis, so that a component becomes infinite and undefined. This usually arises from an error in formulating the model. Conversely, if synthesis cannot keep up with degradation, a component may become zero, which may not be an error. What is required, is to determine whether any components become infinite at steady state and, if not, to determine the steady-state values of the components in terms of the labels.

Elimination also takes a different form in (21) because it is non-homogeneous: if *x* is a steady state of (21), it does not follow that 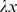 is also a steady state. Indeed, the single degree of freedom that is found in a strongly-connected graph at steady state is no longer present in the partial graph, as the total amount of matter in the system is no longer conserved. Irrespective of how much matter is present initially, it is the rates at which matter enters and leaves the system that determine the final distribution of amounts, if a steady state is reached. This requirement is expressed in a constraint on the steady state. Setting 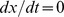 in (21) and using the fact that 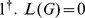 in the core graph, we see that
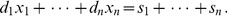
(22)This reflects the fact that synthesis and degradation must be in overall balance if 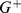 is to have a steady state.

To determine the values of the steady state, construct a labelled, directed graph 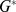 by adding a new vertex 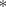 to *G* (Figure 4B). For each of the partial edges above, introduce into 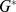 the edges
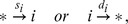
respectively. 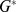 is a proper labelled, directed graph, whose Laplacian dynamics are governed by (3). This graph enables a complete solution to the problem raised above. We focus on the case that is most relevant to the applications by assuming that 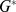 is strongly connected. This will be the case if the core graph *G* is strongly connected and there is at least one synthesis edge and one degradation edge. However, 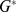 may be strongly connected even when *G* is not (Figure 4A, B), so that this analysis applies to a wider class of graphs than previously.

If 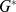 is strongly connected, then the MTT provides a basis element for the kernel of the Laplacian, 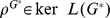. We have annotated 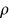 with the superscript 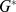 to emphasise that it is quite different from the corresponding basis element, 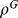, if the core graph also happens to be strongly connected. When 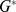 is strongly connected, no components of the partial graph 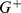 become infinite and 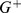 has a unique steady state *x* given by
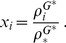
(23)Here, all the vertices of 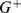 have positive amounts at steady state. The single degree of freedom in 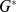 has been used in (23) to ensure that 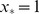. It can easily be checked that 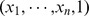 is a steady state of 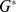 if, and only if, 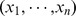 is a steady state of 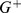. The condition for vertex 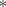 to be at steady state in 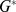 with 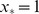 corresponds exactly to equation (22) for synthesis and degradation to be in balance in 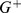.

Equation (23) shows how synthesis and degradation can be readily accommodated within the linear framework. It may be used to revisit all the examples discussed previously to understand the impact of synthesis and degradation. It also opens up for analysis a range of new biological examples. For instance, regulated degradation is a key mechanism in the Wnt/beta-catenin and death-receptor signalling pathways, [71]–[73]. Analysis of these using the linear framework is work in progress.

## Discussion

We have shown that a simple, linear, graph-theoretic framework integrates time-scale separation analysis across many different biological areas. An expert in one of these application areas might say that we have not added anything new to that particular area. The expert would have a point. However, the literature suggests that experts in different areas are apparently unaware that they are all doing the same thing. The aim of this paper has been to reveal this shared framework and to clarify its essentials. The neutral mathematical language adopted here–graphs, spanning trees, strong-connectivity–is spoken more widely than the dialect adopted in any particular area, allowing a broader community access to the ideas. The key insight of the paper is that *elimination of internal complexity is a linear procedure that works because the underlying graph is strongly connected*. To the best of our knowledge, this has not been articulated previously nor has it been made evident how broadly this idea can be applied.

We believe significant advantages accrue from using such a framework. First, as mentioned, it helps break down the barriers between areas: a technique developed in one area may be exploited in many others. Second, the framework reveals the simplicity that is obscured by contextual details. Third, the quantitative analysis of biochemical systems acquires a foundation, instead of appearing as a series of *ad hoc* calculations. Fourth, such a foundation permits new kinds of analysis, such as incorporating synthesis and degradation, that can now be used wherever the framework can be applied.

The framework not only unifies, it also suggests new problems to explore. An intriguing question is whether the dynamical behaviour of a sub-system retains any vestige of the elimination that becomes feasible at steady state. When a graph is strongly connected, the many degrees of freedom that are present at the start of the dynamics (equivalent to the number of vertices in the graph) collapse to a single-degree of freedom at steady state. But how does this collapse come about over time? Do the degrees of freedom gradually “condense”? If so, what does this process of condensation reveal about the architecture of the graph? It is well known that transient dynamics, prior to reaching steady state, are informative about rate constants but it is conceivable that the way in which degrees of freedom are lost may also tell us about the structure of the graph and, thereby, about the structure of the underlying network of biochemical reactions.

Time-scale separation is often used to simplify the dynamics of the slow components. In the Michaelis-Menten example, for instance, the differential equation in (2) is a simplified description of the system’s behaviour, in terms of the slow components only. The method of “singular perturbation” provides a systematic way to determine the quality of this approximation and to understand how it depends on the separation between the time scales, [74]. This has been undertaken for the Michaelis-Menten reaction mechanism, [75], [76], but, surprisingly, it appears not to have been investigated further in enzyme kinetics and not at all in other biological areas. The linear framework provides the starting point from which to apply singular perturbation. The key to doing so is to identify one or more non-dimensional parameters, which become small when the time-scales are separated, [77], from which the accuracy of the simplified dynamics can be systematically determined. If such an analysis can be undertaken in generality, then we would have a powerful new tool for simplifying and approximating the dynamical behaviour of complex biochemical systems.

What emerges from this may be a new approach for dealing with molecular complexity. The examples discussed here, especially gene regulation and post-translational modification, are combinatorial mechanisms with the potential for creating astronomical numbers of internal cellular states. Making sense of this combinatorial explosion remains one of the most significant and intractable challenges facing integrative systems biology. It presents particular difficulties for computational methods based on numerical simulation because they require prior specification of all the details of the system, including the number of sites of modification or binding, as well as the values of all rate constants. This has made it hard to distil general principles–those that hold irrespective of such details. The framework introduced here, in contrast, allows these details to be treated symbolically and mathematically, no matter how many components are present. Details, such as the number of modification or binding sites, may be treated as variables, without having to give them numerical values, [46]. A new kind of analysis becomes feasible which may have the potential to rise above the combinatorial explosion.

## Methods

The conclusions were reached by mathematical analysis, for which further details are provided in Supporting Information S1.

## Supporting Information

Supporting Information S1 **Additional mathematical details.** 1 “Kernel of the Laplacian for a general graph”; 2 “Ligand binding at thermodynamic equilibrium”; 3 “Synthesis and degradation”; and the figure “Spanning trees for the labelled directed graph in Paper Figure 4B”.
(PDF)
