# Correction: A Linear Framework for Time-Scale Separation in Nonlinear Biochemical Systems

**DOI:** 10.1371/annotation/fa4c5f9f-4071-4b32-864f-b82c2e4e973b

**Published:** 2013-06-24

**Authors:** Jeremy Gunawardena

There were errors in the typesetting of equations in the article. This file contain corrected versions of these equations: 


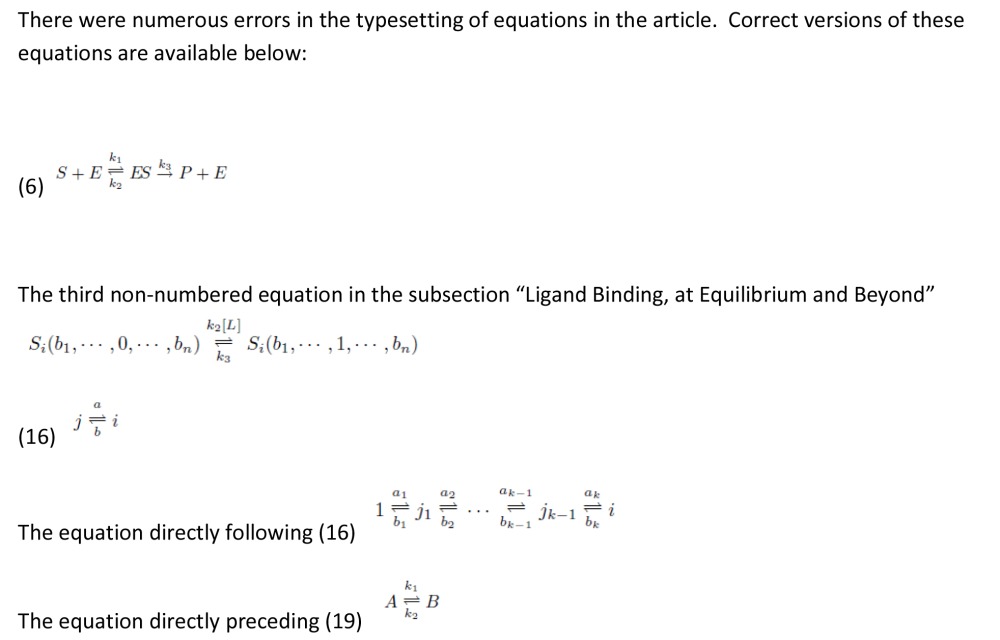


In addition, in the paragraph immediately preceding Equation 23, the first line of the second sentence should have began with the text, "We have annotated ρ with the superscript..." 

