# Correction: A Linear Framework for Time-Scale Separation in Nonlinear Biochemical Systems

**DOI:** 10.1371/annotation/6830fba7-6e52-48f9-9f55-75ee37c75b5a

**Published:** 2013-12-03

**Authors:** Jeremy Gunawardena

This article has been republished to address equation and symbol formatting errors and errors in figure citations. This republication addresses the errors referenced in the previous formal correction. The publisher apologizes for the errors. 

