# Supplementary material for: A Linear Framework for Time-Scale Separation in Nonlinear Biochemical Systems
Source: PLoS One. 2012 May 14;7(5):e36321. doi: 10.1371/journal.pone.0036321 (PMC3351455; doi:10.1371/journal.pone.0036321)
Supplement: Supporting Information S1 — Additional mathematical details. 1 “Kernel of the Laplacian for a general graph”; 2 “Ligand binding at thermodynamic equilibrium”; 3 “Synthesis and degradation”; and the figure “Spanning trees for the labelled directed graph in Paper Figure 4B”. (PDF) [file pone.0036321.s001.pdf]

SUPPORTING INFORMATION

Jeremy Gunawardena

Department of Systems Biology, Harvard Medical School
200 Longwood Avenue, Boston, MA 02115, USA

E-mail: jeremy@hms.harvard.edu

Abstract

This note provides supporting information for the paper *"A linear framework for time-scale separation in nonlinear biochemical systems"*. It should be read in conjunction with the paper. The material is roughly organised in the order in which it appears in the paper but the table of contents provides more information.

Contents

1 Kernel of the Laplacian for a general graph **1**

2 Ligand binding at thermodynamic equilibrium **2**

3 Synthesis and degradation **4**

1 Kernel of the Laplacian for a general graph

We sketch a proof of Paper Equation (5) which gives a basis for the kernel of the Laplacian. While the essential ideas are introduced we leave it to the reader to fill in some of the details.

Let G be an arbitrary labelled, directed graph on the vertices, $1, \cdots, n$. As always, we assume that G has no self loops. Choose $x \in \ker \mathcal{L}(G)$. Let $\overline{G}$ be the acyclic directed graph on the strongly connected components (SCCs) of G, as in Paper Figure 2C. Suppose that the vertices of $\overline{G}$ are $c_1, \cdots, c_m$ and that c_1 is an initial SCC that is not also terminal. By construction, there must be some vertex, $i_1 \in c_1$, with an edge leaving c_1, $i_1 \rightarrow k$, where $k \notin c_1$. If $x_{i_1} > 0$, there is a positive flux of material along this edge. For x to be a steady state, this flux must be balanced by some flux coming into i_1. This can only arise from some edge $i_2 \rightarrow i_1$ with $x_{i_2} > 0$. Taking all such vertices, recursively, yields a subset of vertices that can be the only source of the balancing flux into i_1. However, because i_1 is an initial SCC, this subset is entirely contained in c_1. This SCC has only a limited amount of starting material and therefore cannot indefinitely balance the outgoing flux on the edge $i_1 \rightarrow k$. It follows that $x_{i_1} \leq 0$. However, if $x_{i_1} < 0$ then there is positive flux coming into i_1 along the edge $i_1 \rightarrow k$. This can only be balanced by an edge $i_3 \rightarrow i_1$ with $x_{i_3} < 0$. Arguing recursively in a similar way as above yields a similar contradiction. We conclude that $x_{i_1} = 0$. But then $x_j = 0$ for any vertex j with $j \rightarrow i_1$. Since c_1 is strongly connected, it is then easy to see that $x_j = 0$ for any $j \in c_1$. It follows that x has no support on any initial SCC that is not also terminal. (The support of x is the subset of vertices, i, such that $x_i \neq 0$.)

It is now easy to argue by induction over those SCCs that are not terminal to show that the support of x contains only vertices that are in terminal SCCs. Consider each terminal SCC, t, as a labelled, directed graph, G_t, in its own right, in isolation from the rest of G. Assume that G_t has n_t vertices. Let $x^t \in \mathbb{R}^{n_t}$ be the vector obtained from x by restricting x to those vertices lying in t. Since x has no support outside the terminal SCCs and there are no edges between the terminal SCCs, it should be clear that $x^t \in \ker \mathcal{L}(G_t)$. Let $v^t \in \mathbb{R}^{n_t}$ is the vector coming from the MTT applied to G^t. Since t is strongly connected and $\dim \ker \mathcal{L}(G_t) = 1$, it must be that $x^t = \lambda^t v^t$, for some $\lambda^t \in \mathbb{R}$. Now let $\rho^t \in \mathbb{R}^n$ be the vector constructed in the Paper,

$$(\rho^t)_i = \begin{cases} (v^t)_i & \text{if } i \in t \\ 0 & \text{otherwise.} \end{cases}$$

Since the terminal SCCs are disjoint, the vectors, $\rho^1, \cdots, \rho^T$, are linearly independent by construction. Evidently, $x = \sum_{t=1}^{T} \lambda^t \rho^t$. Hence, these vectors form a basis for the kernel of the Laplacian,

$$\ker \mathcal{L}(G) = \langle \rho^1, \cdots, \rho^T \rangle ,$$

which proves Paper Equation (5).

2 Ligand binding at thermodynamic equilibrium

Suppose that we have a graph, G, constructed as described in the section of the Paper on "Ligand binding, at equilibrium and beyond". We assume that the vertices are enumerated, $1, 2, \cdots$, omitting the details of conformation and ligand binding, and that the reference vertex 1 corresponds to a microstate in which no ligands are bound. The labels are either rate constants, corresponding to conformational transitions or ligand unbinding events, or expressions of the form $k[L]$, corresponding to the binding of ligand L with rate constant k. We assume that the system is at thermodynamic equilibrium, so that every edge is reversible. As long as G is connected, which we assume, it is automatically strongly connected.

We want to show that the cycle condition holds on G if, and only, if every equilibrium state satisfies DB. Recall that an equilibrium state, x, satisfies detailed balance (DB) if every pair of reversible edges

$$j \underset{b}{\overset{a}{\rightleftharpoons}} i$$

is independently at equilibrium, irrespective of any other edges in which the vertices play a role. It follows that,

$$x_i = Kx_j \,, \tag{1}$$

where $K = a/b$ is the equilibrium constant. The cycle condition on G states that, for any cycle of reversible edges, the product of the rate constants on the edges going clockwise is equal to the product of the rate constants on the edges going counterclockwise.

Suppose first that every equilibrium state satisfies DB. Choose an equilibrium state, x. Since DB is satisfied, the net flux through any reversible edge is zero. Hence, the net flux around any cycle of reversible edges is also zero. Choose any such cycle and pick any two vertices on it, say i' and j'. The cycle can be broken into a pair of directed paths between i' to j'. Applying (1) repeatedly on each path gives two expressions for $x_{j'}$ in terms of $x_{i'}$, as in Paper Equation 17. Equating these expressions, cancelling ligand concentrations and clearing denominators, yields the cycle condition. Since the cycle was chosen arbitrarily, this proves the "if" part.

Now suppose the cycle condition holds. Let x be any equilibrium state. We need to show that x satisfies DB. We construct an alternative steady state y, which we show to satisfy DB, and then prove that $y = x$. Recall that the reference microstate, 1, has no ligands bound and set $y_1 = x_1$. For any other microstate j, choose some path of reversible edges from 1 to j, which must exist since G is strongly connected, and use (1) to express y_j in terms of y_1, as in Paper Equation 17. Now choose some other path from 1 to j and obtain a second expression for y_j in terms of y_1. The two paths together form a cycle of reversible edges, to which the cycle condition applies. Reorganising the cycle condition and putting in the appropriate ligand concentrations shows that the two path expressions give the same result for y_j. Hence, this quantity is well defined, irrespective of the path chosen.

We have unambiguously defined a state, y, of G but we have yet to show that it is a steady state. Consider any reversible edge between the microstates i and j. Choose a pair of reversible paths from 1 to i and from 1 to j. Together with the reversible edge between i and j, this gives

a cycle of reversible edges. Applying the cycle condition, it is easy to see that, in the state y, the reversible edge between i and j must be independently at equilibrium. This not only implies that y is a steady state but also that y satisfies DB. But now, G is strongly connected and so $\dim \ker \mathcal{L}(G) = 1$. Hence, $y = \lambda x$ for some $\lambda \in \mathbb{R}$. Since $y_1 = x_1$, $\lambda = 1$. Hence, $y = x$ and therefore x satisfies DB. This completes the proof.

For applications of the framework at equilibrium, it is necessary to calculate various aggregate measures, as shown in Paper Figure 3. We briefly discuss here various techniques for calculating fractional saturation, which is widely used to quantify allosteric enzyme activity.

Provided the reference vertex, 1, has no ligands bound, as we have assumed, then, in any steady state x, the quantity x_i/x_1 is a monomial in the ligand concentrations and the power to which $[L]$ appears is the number of L molecules bound in microstate i. Hence, the concentration of states in which L is bound is given by

$$[L](\partial x_{tot}/\partial[L])$$

and the fractional saturation, or average concentration of states bound by L, is the logarithmic derivative,

$$\left(\frac{[L]}{x_{tot}}\right)\frac{\partial x_{tot}}{\partial[L]} \,. \tag{2}$$

More complex aggregate concentrations can be worked out in a similar way.

The calculation of x_{tot} needed in (2) can be simplified by suitably decomposing the graph, as illustrated by the sum and product formulas below.

DB implies that any equilibrium state x^G of G gives, by restriction, an equilibrium state x^R of any subgraph, R. If R and T are subgraphs that are disjoint (no vertex in common), which together span G, we get the sum formula

$$(x^G)_{tot} = (x^R)_{tot} + (x^T)_{tot} \,. \tag{3}$$

If ligands bind independently, so that the site-specific rate constants are independent of the microstate in which ligand binds, then the graph may be decomposed into a product of the graphs for single site binding. The product of two graphs is defined as follows. Suppose that G is a labelled, directed graph on the vertices $g_1, \cdots, g_n$ and that H is a labelled, directed graph on the vertices $h_1, \cdots, h_m$. The product $G \times H$ is the labelled, directed graph on the vertices $g_i \times h_j$ in which there is a labelled edge

$$g_{i_1} \times h_{j_1} \xrightarrow{a} g_{i_2} \times h_{j_1}$$

whenever there is a labelled edge $g_{i_1} \xrightarrow{a} g_{i_2}$ in G and, symmetrically, there is a labelled edge

$$g_{i_1} \times h_{j_1} \xrightarrow{b} g_{i_1} \times h_{j_2}$$

whenever there is a labelled edge $h_{j_1} \xrightarrow{b} h_{j_2}$ in H. There are no edges in $G \times H$ other than these. This construction captures the fact that a change in state of either factor is independent of the state of the other factor.

The equilibrium state of a product may be obtained from those of its factors as follows. Define the normalised total by $\pi(G) = x_{tot}/x_1$, where x is any equilibrium state. It follows from Paper Equation 4 that $\pi(G)$ is independent of x, although it may depend on the choice of reference vertex. With 1×1 as the reference in $G \times H$, it is not difficult to prove the product formula,

$$\pi(G \times H) = \pi(G) \times \pi(H)\,. \tag{4}$$

Independent binding allows $\pi(G)$ to be factorised.

Formulas (2), (3) and (4) are helpful for typical calculations at thermodynamic equilibrium.

3 Synthesis and degradation

For the partial graph, G^+, in Paper Figure 4A, the corresponding labelled, directed graph, G^*, in Paper Figure 4B is strongly connected, with the Laplacian shown in Paper Figure 4D. Applying the MTT leads to the spanning trees in Figure 1, from which the components of the basis element $\rho^{G^*} \in \ker \mathcal{L}(G^*)$ can be read off according to the prescription in Paper Figure 4, to give

$$\begin{aligned}
\rho_1^{G^*} &= a_1s_2d_2 + a_4s_1d_2 + a_1s_1d_2 \\
\rho_2^{G^*} &= a_2s_1d_2 + a_3s_2d_2 + s_2d_1d_2 + a_2s_2d_2 \\
\rho_3^{G^*} &= a_1a_3s_2 + a_3a_4s_1 + a_2a_4s_1 + a_4s_2d_1 + a_3a_4s_2 + a_1a_3s_1 + a_2a_4s_2 \\
\rho_*^{G^*} &= a_1d_1d_2 + a_4d_1d_2 + a_3a_4d_2 + a_1a_3d_2 + a_2a_4d_2\,.
\end{aligned}$$

The unique steady state of G^+ can now be calculated from Paper Equation 23.

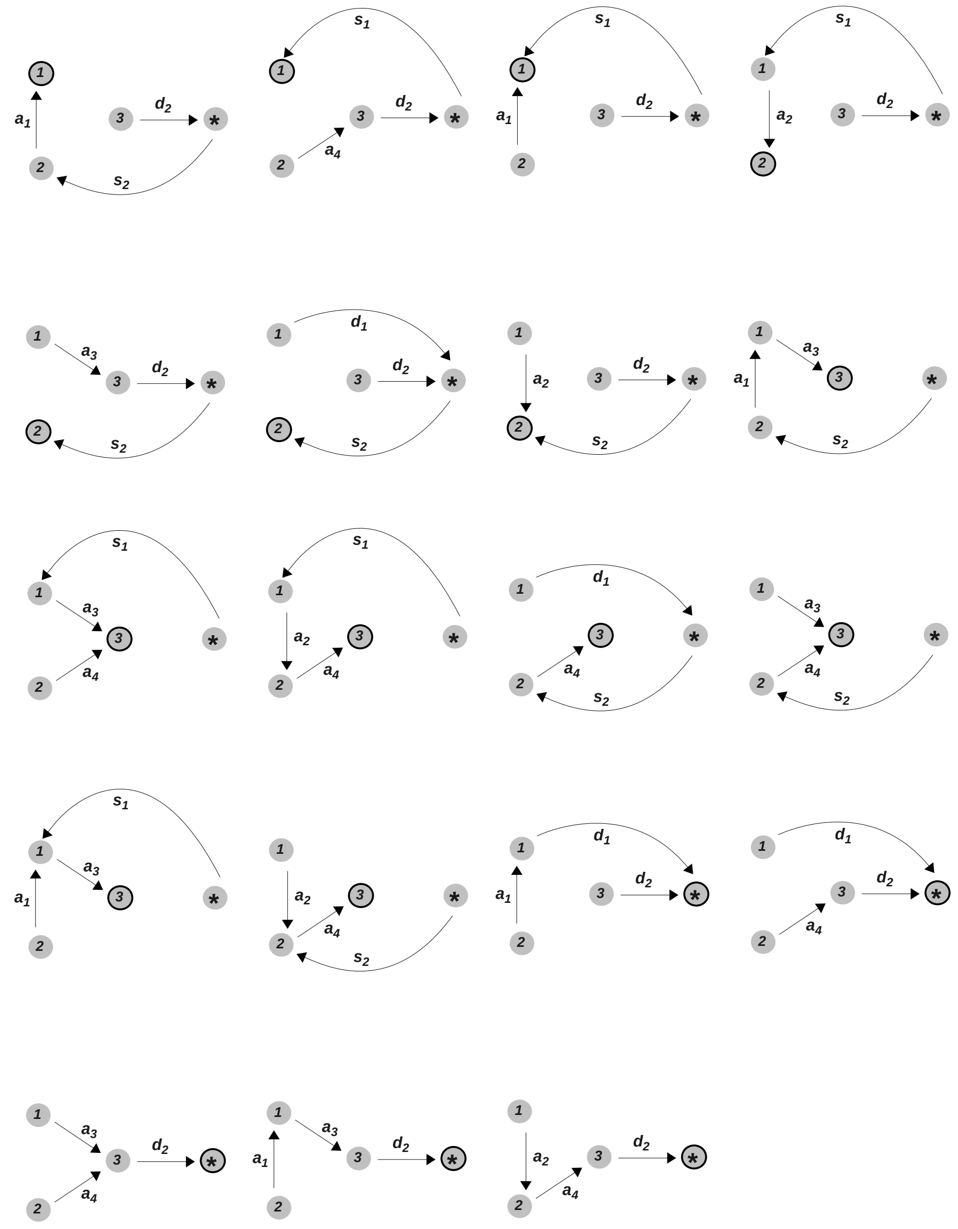

Figure 1: Spanning trees for the labelled, directed graph in Paper Figure 4B. The 19 trees are listed, with each root indicated by a black circle around the corresponding vertex.
